# A Longitudinal Qualitative Analysis of the Way Peer Support Specialist Roles Change Over Time in a Psychiatric Hospital Setting in Asia

**DOI:** 10.1007/s10488-021-01158-y

**Published:** 2021-08-14

**Authors:** Daniel Poremski, Jonathan Kuek, Yuan Qi, Ziqiang Li, Kah Lai Yow, Pui Wai Eu, Hong Choon Chua

**Affiliations:** 1 Health Intelligence Unit, Institute of Mental Health, Buangkok Green, Singapore, Singapore; 2 Research Department, Institute of Mental Health, Buangkok Green, Singapore, Singapore; 3 Department of Nursing, Institute of Mental Health, Buangkok Green, Singapore, Singapore; 4 Department of Allied Health Services, Institute of Mental Health, Buangkok Green, Singapore, Singapore; 5 Department of General Psychiatry, Institute of Mental Health, Buangkok Green, Singapore, Singapore; 6 Office of the CEO, Institute of Mental Health, Buangkok Green, Singapore, Singapore

**Keywords:** Peer support, Service maturation, Longitudinal qualitative methods, Career trajectories

## Abstract

The current study seeks to determine how peer support roles change as peer support specialists’ positions within organizations and departments mature. We followed ten peer support specialists over the course of a year, interviewing them at three points, starting approximately three months after they began working as peer support specialists. We used an inductive process to analyze our data and followed guidelines on the structuring of longitudinal qualitative trajectories to divide the data into watershed moments. Our participants worked in a variety of departments in the hospital, and their service use experiences generally echo those of their service users. Participants appear to pass through four phases over the course of their employment as peers: early beginnings, establishing the role, role narrowing, and role sustainability. Services wishing to integrate new peers must be aware of the time required for integration. Having general job descriptions limited to specifying that peers are expected to use their lived experience to support current service users may lead to uncertainty amongst new and existing staff. Without role clarity, peers may struggle to find their place. Pairing new staff with mentors may limit this burden. As roles consolidate, boundaries may emerge. If these boundaries narrow the role of the PSS, they may no longer find the role appealing. They may then choose other caregiver roles with wider or different spheres of influence. Organizations may benefit by clearly indicating if they expect peer support positions to be static or transitionary.

## Introduction

Personal recovery and recovery-oriented care models have slowly but steadily gained traction in many western countries during the past three decades. This paradigm shift is congruent with the overt commitments and declarations made by the American Surgeon General David Satcher in his 1999 landmark report on the need to redefine mental health service models (Davidson, 2016). Since then, a multitude of recovery-oriented models of care have been proposed. Of prominence was the formal introduction of peer support workers, which were seen as the personification of such recovery-oriented models. These models rely on people with lived experience of mental illness to provide support to others currently experiencing mental illness during their recovery journey (Perkins & Repper, 2019; Repper & Carter, 2011; Salzer et al., 2010). These types of service providers are called peer support specialists (PSS) and obtain their professional qualifications after their experiences of mental illnesses, differentiating them from service providers in recovery.

Peer support service models have demonstrated effectiveness in a variety of settings (Lloyd-Evans et al., 2014; White et al., 2020) including supported employment settings (Kern et al., 2013), when engaging difficult to reach populations, namely, individuals living in rural areas (Cheesmond et al., 2020); and in supporting the management of specific mental health conditions; for example, substance use disorders (Humphreys et al., 2004), borderline personality disorders (Barr et al., 2020), and severe mental illnesses (Davidson et al., 2012; Fan et al., 2018; Fortuna et al., 2020). Reviews and qualitative research support these findings of effectiveness and highlight the potential for peer support services to achieve recovery outcomes to the same degree as those seen in traditional mental health services. (Davidson, 2016; Davidson et al., 2006, 2012; Ibrahim et al., 2020; King & Simmons, 2018; Shalaby & Agyapong, 2020).

More recently, research has focused attention on understanding and carefully unpacking the various mechanisms that contribute to the effectiveness of peer support work (Gillard, 2019; King & Simmons, 2018; Simmons et al., 2020). For example, a review of mechanisms that underpin peer support work highlighted five core aspects—their lived experience, love labor, the liminal position peer workers hold (i.e., a state where peer support staff are neither fully staff members nor consumers and operate in the space between the two roles allowing for more authentic partnerships to be forged), strengths-focused social and practical support, and the helper role (Watson, 2019). An earlier study which sought to develop a model to explain how PSS created change also identified similar mechanisms such as the building of trusting relationships based on a shared lived experience, role modelling of recovery, and engaging them within the community (Gillard et al., 2015). Additionally, a consultation commissioned by Together in the United Kingdom highlights several challenges to the professionalization of peer support services such as lack of financial support, inappropriate content of existing training programs, and misalignment with existing organizational structures (Faulkner & Kalathil, 2012). Indeed, there is some literature that suggests this lack of understanding has resulted in several negative consequences such as lack of further training, low pay, and discrimination or prejudice from non-peer workers (Adams, 2020; Jones et al., 2020; Kuek et al., 2021). Such literature helps policymakers implement new services designed to maximize their success and sustainability.

This evidence, however, does not adequately address how the PSS role within an organization evolves or adjusts to the specific setting. Simmons et al. (2020) attempted to address this gap in the knowledge pertaining to PSS services offered to youths by following their career development. They noted that PSS experience three distinct phenomenon that helped them adapt to their new context. Their participants noted that the value of their lived experience became more salient as their experience with it as a tool for recovery matured and that roles became clearer over time, echoing results of similar research (Otte et al., 2020). Simmons et al. (2020) noted as well that the overall experience was characterized by a shift to greater hope about themselves. Instilling hope is an essential element of PSS work (Chinman et al., 2016; King & Simmons, 2018) and such qualitative longitudinal analyses demonstrate that PSS do not necessarily begin their role with all the traits necessary to be effective PSS, but rather that they may emerge with time.

### Peer Support Services in Asia

Services that have been implemented in Asian contexts, such as those in Hong Kong, have shown modest impacts (Chang & Liu, 2014; Tse et al., 2013). However, other Asian nations have been slow to embrace the movement. In China and India the feasibility of such peer support work has only recently been the focus of dedicated research (Yunge Fan et al., 2019; Pathare et al., 2018; Yu et al., 2018). While authors note the potential benefits of peer support ideologies, they also note the cultural barriers and accompanying lack of investment. A recent scoping review identified that people in Asia tended to have a very medicalized view of mental health conditions (Kuek et al., 2020). Correspondingly, people hold pragmatic opinions of recovery, preferring a more functional medical approach to those described in personal recovery models (Kuek et al., 2020), such as those on which peer support ideologies depend. In these settings it may be difficult for people to assume professional or social roles that require them to openly identifying as someone who has experienced mental illness. This mindset could influence the way PSS are received and hence limit their effectiveness.

In Singapore, a small nation populated by Chinese, Indian and Malay ethnicities, arguments exist to attempt to generalize the potential positive impact of peer support services. These relate mostly to the treatment models implemented in its healthcare system, which strongly resemble systems in the UK and the US. However, counterarguments also exist. As in other Asian nations, stigma surrounding mental illness persists (Yuan et al., 2016). These arguments remain theoretical, and local knowledge is required to help guide relevant policies. To that end, it is crucial we carefully understand and examine how peer support workers are integrated within a new setting. Thus, our primary goal was to explore the changes and evolutions to the peer support role, if any presented themselves, within a tertiary psychiatric hospital setting in Singapore.

## Method

We decided to answer our research question by conducting repeated qualitative interviews in a longitudinal study design that followed the  design outlined by Grossoehme and Lipstein (2016). The study is nested in a larger mixed-methods quasi-experimental study designed to shed light on the overall impact of peer support services on the health of the peers and on the health of their service users. We followed the Consolidated criteria for reporting qualitative research checklist (Tong et al., 2007) to guide the preparation of this paper. Institutional (#646-2018) and national ethic review committees (#2018/01131) approved the study. All participants gave written informed consent at the first interview. Subsequently, we sought verbal consent before each follow-up interview to ensure participants were still willing to participate.

### Setting

The host institute, Institute of Mental Health (IMH), is the largest source of tertiary psychiatric care in Singapore. It serves the multi-ethnic nation of approximately 5.7 million. It has approximately 1900 inpatient beds, of which half are for acute care. It provides services across the illness journey and across all age groups. Peer support services have existed for several years in IMH, but only in certain departments, such as those treating addiction, first episode psychosis, and mood disorders.

Since 2016, a tripartite alliance of institutes in Singapore has offered training and certification of people with lived experience to develop the peer support segment of the local healthcare workforce and integrate them into various roles across Singapore. The training consists of 80 h of classroom-based coursework. The course was developed with an American service provider focused on recovery practices and peer support training. The curriculum covers common service provider topics, such as setting boundaries and managing crises, service user topics, such as recognising stressors and coping with them, and with peer support topics, such as crafting narratives, and managing disclosure. The course also prepares PSS for the interactions they may have with staff. Knowledge is tested via standard pedagogical means. The coursework is followed by three months of supervised placement in a peer support role with supervision from the course instructors and experienced PSS managers. This placement is designed to provide PSS with greater exposure to the specific position into which they may be hired, but it is not possible for the training to provide exposure to all the potential situations in which PSS may find themselves. While there was no mentoring when study participants completed their certification, the PSS community has a tight-knit community of practice where information is shared.

In 2017, IMH began systematic efforts to integrate PSS into several inpatient and outpatient departments including psychosocial rehabilitation, case management, community-based services, occupational therapy, inpatient, outpatient and emergency services. To this end, wards and departments willing to place PSS were identified, and subsequently prepared for the placement of PSS. This was done through various means including presentations from UK and US recovery and PSS specialists, presentations from current PSS. It must be noted that while the organization expanded recovery-oriented training, which did elaborate on the role of PSS, staff managing PSS did not receive individual-level PSS-specific training. As IMH is the largest source of psychiatric care in Singapore, it is also the largest single-site employer of PSS, with a workforce of 18 PSS as of December 2020.

The job descriptions of the PSS vary between departments, but universally include service seeker/user supportive roles based on the principle that lived experience of illness and recovery should be used to help current service users achieve their goals. All PSS operate as part of a wider clinical team, instead of independently. They are also all engaged in continued supervision with their managers, in accordance to fidelity measures (Chinman et al., 2016). Because of the wide variety of settings in which PSS have been integrated, it is difficult to provide specific details about their routine activities. This general description allows individual departments to set employment duties to tailor activities to the needs of department-specific service users. Accordingly, a PSS working on the acute inpatient ward would be conducting different activities than those integrated into the community health teams. The common denominator in every case is that people have general experience with the program into which they are placed and are tasked with using their lived experience to help current service users understand their services, their condition, and their path to recovery. At this point it is relevant to note that while there is an alignment of lived experience with the setting in which PSS work, efforts are made to avoid placing PSS into wards or divisions in which they may have previously received treatment.

### Sampling and Recruitment

We have defined PSS as the people with lived experience of mental illness who gained their training and certification after experiencing mental illness, consequently they tend to be further along their recovery journey. They are also salaried employees who work under a formal mandate as part of a multidisciplinary treatment team. They are not engaged in mutual support activities. In addition to these criteria, PSS were eligible if they had completed their probation period at the hospital. Community-based PSS were not included because the focus of our study was to understand the PSS role within the hospital. It is also for this reason that we did not consult PSS who were unsuccessful in completing their probation period (n = 1).

We recruited PSS after their probation period, approximately three months from joining the organization. We attempted to recruit as many PSS without placing undue pressure on those who declined. As there were relatively few PSS who joined the institution after this study began, we also recruited individuals who were already working at the hospital as PSS, excluding only those who had worked at the hospital for more than two years. Before this period, the hospital had not pursued extensive PSS placement, and therefore PSS who had been with the hospital for longer than two years would have had different experiences. We recruited a final sample of ten PSS, as seven others were not eligible, and one declined participation.

### Interview Process

We interviewed participants three times, separated by four-month gaps (baseline, 4 months, and 8 months) between April 2019 and April 2020. The appendix contains a copy of the general interview guide for reference.

We used a theoretical framework derived from a previous study examining service fidelity (Chinman et al., 2016) to structure the interview. The framework provided information on key aspects of program fidelity (e.g., activities peer specialists engaged in, documentation processes, resources they were given). Accordingly, our interview guide referenced the fidelity questions, which related to these broad domains. The emphasis on program fidelity in the peer support literature has been relatively weak (Gillard, 2019) but is justifiably growing (King & Simmons, 2018). This framework also allowed us to determine if and when elements associated with high fidelity PSS developed over participants’ careers.

The first interview focused on establishing a baseline understanding of the PSS and their recovery. We also discussed their trajectory to peer support work. The latter two interviews focused on understanding how their role as a PSS shifted over time. The same interviewer, the first author, conducted all interviews to allow participants to be at greater ease and allow for the formation of a psychologically safe interview space. Though interviews were audio-recorded, the interviewer actively took notes throughout the interviews, and reflected upon the content of each interview after each session.

### Reflexivity

All members of the research team had some form of interest in the recovery and PSS movements in Singapore, but none of the authors stand to gain or lose from the outcomes of this study. We are aware of the potential bias to only capture favorable results while exploring how the roles have changed. However, as our interest pertains to the improvement of services, we understand that succumbing to this attention bias prevents potential gaps or shortcomings from being identified. Furthermore, the lead author, who was also the interviewer, was not involved in the management of the PSS. This fact was made known to the participants from the beginning. Hence, we believe participants did not feel pressured into responding in a way that would glorify the program or feed program administrator bias.

It is also important to note that the interviewer, while not of the same culture as the participants, was of similar age and has extensive experience conducting longitudinal interviews with multi-ethnic populations that vary in socioeconomic status. On average across the three time points, interviews lasted 63 min (SD 20 min), slightly above the allotted time. Participants spoke for an average of 67.8% (SD 15.2) of the time. While these metrics are not proxy indicators of power balance, we submit them to the reader to suggest that participants felt comfortable enough during the interviews to speak at their ease and were willing to devote extra time to their participation.

### Analyses

To make the most use of the repeated interviews, we followed suggestions offered in the literature (Grossoehme & Lipstein, 2016) and ordered content into phases. We analyzed interviews and notes to develop potential timelines as the interviews progressed and in the interim between the follow-up points. This resembles a constant comparative approach commonly, but not exclusively, used in grounded theory.

The second author transcribed interviews verbatim shortly after interviews were completed. We made light grammatical corrections only to the quotes presented below. The first author led the coding process, which was conducted as the interviews progressed. The first and second authors began by partitioning the content of the interviews to allow the team to focus on the various topics covered during the interviews. We worked simultaneously to establish codes over the course of the entire data collection period. The first author established exemplar quotes to aid the team in the coding and periodically reviewed the coding of others. The team discussed the codes and agreed upon their meaning as the study progressed. When new codes were created, or when codes were revised, previously coded interviews were recoded with the help of the software. The second author coded the bulk of the content, while the first author independently refined larger codes to develop meaningful sub-codes. We cross-coded several codes to allow their overlaps to emerge. For the purpose of the present project, we relied on content coded to match with the phases of employment. The goal of conducting multiple qualitative interviews was to obtain an idea of how things changed to help us develop a better understanding of trajectories. Unlike single interviews, which provide a somewhat distant and detached view of the past and the future, repeated interviews can more clearly treat events, once as expected developments and the next as completed developments. This repetition allowed us to treat expectations as something that might be built upon in the following interview. The multiplicity of the repeated interviews also allowed participants to develop a narrative skill over time. While it is prudent to ask participants to prepare for an interview, in repeated interviews, participants become accustomed to talking about their narrative with an eye to identifying change and not simply fact. While it might be argued that PSS by the very nature of their profession become skilled storytellers, the purpose of qualitative interviews is not the same as the purpose of sharing lived experience. We used NVivo 11 (NVivo 11 Plus, 2017) to facilitate the analysis.

## Findings

Our sample consisted of ten PSS. All scheduled follow-ups were completed, leading to a dataset with 30 individual interviews. One participant left his role over the course of the study; his last interview was not recorded, following his wishes. In this case, the interviewer supplied the interview questions prior to the interview, and the participant wrote out his answers prior to the interview to come prepared for the interview.

Their average age was 30 (SD: 6.2), six were women, and four were men. Their ethnicities reflected those of Singapore, with representation from Chinese, Malay and Indian groups. They also described reaching post-secondary education. While these traits suggest PSS were younger than the average hospital service user, achieved higher level of education, and they include more women, their mental health and service experiences, by virtue of the way they are selected and placed within the hospital, resemble those of their service users. For example, a PSS with experience of depression would be placed in the department of mood and anxiety, and not the department of first episode psychosis.

None were admitted during the follow-up period or within the year preceding their employment as PSS. Their experiences of how being a PSS impacted their health is published elsewhere.

We organized our data into four phases (early beginnings, establishing the role, role narrowing, and role sustainability) that participants appeared to travel through over the course of their narratives and role maturation.

### Early Beginnings

Role confusion was a prominent theme of the early phase of their time as PSS. They were unsure of how they best fit within the multi-disciplinary teams and how they would be perceived by their colleagues who were not in similar positions. Additionally, existing staff members often did not understand what a PSS’ role was intended to be within the teams.

“I think one of the greatest challenge of a peer support specialist work is the clarity of role. The clarity of role always comes into question, it’s always in doubt, because it is intangible at times and it’s hard to quantify our outcomes. So it’s not very straight, black and white kind of thing?”—50004_3

“I guess also the lack of clarity in terms of like what the role of PSS should be. I think the very generic and very basic ones is to use the lived experience to help the peers go through recovery, but I think along the way, we’ve kind of taken on a lot of different hats.”—50005_2

“I think when I first started, I was not very sure about what I was supposed to be doing and all”—50009_2

This was exacerbated as several PSS noted how their role could be very broad, given the nature of the work they had been trained to do. Their supervisors were also unsure of how best to make use of PSS and their unique skillset. The degree of supervision, the degree of accommodation for sick days, and the degree of integration into confidential conversations all came into question and had to be addressed at an individual level. This was likely because departments were given a high degree of autonomy to prepare for the introduction of PSS. Furthermore, while it might have been clear in policy that the PSS were to be integrated in to the clinical teams, the degree to which the practice was adopted appeared to depend more on the personal comfort level of those interacting with the PSS. Participants felt that effective communication and being proactive with their supervisor and the team were essential in clarifying their roles within the group.

“Maybe their supervisors are also confused, so it’s for both of them to think through and have some clarity on A, the role, B, like what they actually want to do, because actually peer support is just very general, very big.”—50004_2

“I think I’ve reached this point where I’m quite clear, in fact I’m very clear about my role, like what I’m supposed to do and there’s this basic structure and I think initially it was just like coming up with things because when you are in a new department and they’ve never had PSS, they’ve never heard of it, you actually suggest or initiate right?”—50009_2

This confusion was exacerbated by perceived similarities between the role of the PSS and the roles of other healthcare professionals.

“Our roles right, okay, if [for example] we bring in like a therapist, it’s very clear cut, counselling, the type of counselling that they’re being trained, 1-to-1, groups, straightforward right? As a PSS, we do so many other things, and we dabble so many variety of things, that sometimes people don’t know where the focus is.”—50004_3

“Kind of like, almost playing like mini case managers, we do a bit of everything. So I think that even that lack of clarity, it makes it hard, because sometimes we might also overstep our boundaries, like doing the work of case managers or even sometimes like a psychologist because we have to do some of this work but we don’t really have a very clear line of saying “oh okay maybe this is where we shouldn’t do this anymore.”—50005_2

While the training PSS received during their certification may have addressed personal boundaries when working with service users, it likely could not fully address the issue of professional boundaries. It was evidently harder for the PSS to set and hold those boundaries in new settings where it might be natural to defer to the decisions of senior members of the team.

### Establishing the Role

The next phase was characterized by progressive role clarity, during which their understanding of their duties and the duties of those around them became concrete and clearly delineated. It was a two-way relationship, whereby PSS also started understanding the roles of their colleagues. This clarity helped reduced tensions between them.

“Okay, so in the past, the role issue still existed, but for now like the issue about the role has changed. It used to be in the past, like if the challenge about the role is more of like, me being a PSS, why am I leading a OT group and what is my role in there, that kind of thing.”—50004_2

“I think I am still able to, I mean I know my role well enough. That is what I feel, and my colleagues also shared that they learned about PSS and the role because I know about my role. And we did not always, when we started, we did not know what the role was going to be like, I was also figuring out how things would go and I didn’t have a clear PSS idea.”—50009_3

Peers who had joined the organization to replace a PSS that had previously worked with the team none-the-less grappled with this issue. In some cases, when the previous PSS had experienced a deterioration in condition, the new PSS faced inherited barriers and negative prejudices. One PSS who replaced a colleague who had relapsed noted that not only did he have to establish his PSS role, but he also had to correct misconceptions that developed amongst staff affected by the deteriorating health of his predecessor.

“Unfortunately, before I came into my position I had one very popular IMH PSS seated in my seat doing my work, unfortunate he has displayed a lot of symptoms was very symptomatic, not taking care of himself, even to the point of 2 weeks ago he was still admitted. And then every, during the phase he worked in he displayed a lot of psychotic symptoms, a lot of people got scared, a lot of people already firmed up an impression about people with a diagnosis working with them, so that was already damaged. […]”—50007_3

Correcting these misconceptions took dedication and patience, but eventually led to colleagues perceiving the PSS’s capacity to work effectively.

“Because I have heard some people “*OH you can do all this? wow, you know before that person [couldn’t]…*” I have heard that tune more than once.”—50007_3

For PSS who joined existing PSS in established roles, the pioneering work of their senior colleague had not gone unnoticed. Such partnerships highlighted the importance of pairing PSS with existing PSS as a method of achieving role clarity. Having a partner to support and guide them during their early days as a new PSS in the hospital provided a sense of comfort and safety.

“Yeah, so it’s like she’s kind of my guardian for that first maybe one or two months. So I was quite well adjusted like to the team and all that. She takes care of me very well and I think that really helps when there is a pair, when we have a pair in the unit. Whereas I think some of the departments here are doing solo work. Sometimes I can be a bit worry for them when it’s like silos.”—50001_1

### Role Narrowing

The next phase was characterized, for some, by perceived role narrowing: as management and co-workers began to understand what PSS should be doing, so too did they begin to understand what PSS should not be doing. Tasks that had originally been open to PSS now fell outside of their domain. As a result, management no longer encouraged the PSS to assume these additional responsibilities, but rather were perceived as directing the focus of the PSS to their core responsibilities.

“But I think recently as well, we’ve kind of also started clamping down on that. I know my supervisors were kind of looking like “*okay is this really PSS work, if it’s not considered PSS work, we shouldn’t be doing it*”. Whereas in the previous years, it was more like “okay we can still kind of like have a learning process coming out from that.”—50005_3

Sometimes these restrictions eliminated task variety. In other situations, these restrictions kept PSS from gaining insight into the way other professions conducted themselves with respect to service users because the restrictions also isolated them from working closely with other types of professionals to, presumably, prevent task drift. In very few cases this role narrowing reduced their opportunity to work with service users. The role narrowing made their jobs less varied, more repetitive, and according to our participants, mundane.

Wanting to be able to do more than their narrow role to influence the care provided by the hospital led them to question what they could do to have a greater impact on the lives of their service users.

“I told my supervisor that “*I know that more can done for this lady, but this is the max that peer support can do, I cannot do any more, but I don’t see the team coming in to meet me here*”. You know what I mean? But I cannot go beyond that.”—50006_2

This sentiment was most pronounced in the people who had joined the PSS role in order to give back to their peers and the community. This narrowing of roles was more than simply butting up against the limits of their role, but represented, as some perceived it, an active attempt to limit their influence. Perceiving the role narrowing in such a way led those who described their job a calling (more than simply an occupation) to consider how they may expand their sphere of influence, especially through advocacy and working with staff and their perceptions rather than with service users. It is for these individuals that moving beyond the role became imperative.

### Moving on, Staying on

Over the course of their role maturation all PSS adopted a strong sense of care-giving responsibility that led them to consider the various options for sustaining and advancing their potential contribution to service users. For three PSS, this led them to consider what they could do for their service users within their role as PSS. These participants did not grapple much with the implications of their role narrowing and did not speak as intensely about their plans for the future. They were satisfied with continuing their duties and contributing to the care of their service users in their current capacity, but looked forward to the development of PSS career pathways within the organization:

“So now is just looking forward. And then from now, why not create steps that we can grow as a PSS profession. Because PSS have been here for more than 5 years. […] Circumstances changes for each person, things get more expensive, living expenses as you live, maybe people you’re taking care of, they will start depending on you, right? So this is also a job at the end of day that we have to think about. So these are actual realities that we have to kind of consider when it has become a profession that we’re so devoted to. So yeah, I think it will be good to have upskilling of PSS simply because when you upskill a PSS, it’s not just for the PSS also, it’s also for the people that they serve. Because then when you upskill your PSS, maybe they’re able to better handle certain situations, better support the peers.”—50002_3

For six PSS, the role restriction led them to contemplate careers outside of PSS that might provide them with new challenges and expanded opportunities to improve the lives of service users. They saw, free from the fog of confusion, that the impact they could have was limited by their position in the service provider hierarchy. Consequently, they followed a path that led them outside of peer roles to transition to jobs that allowed them to help more people, such as counsellors, therapists and case managers.

“I’m actually quite certain that I want to move on from the role. I think [there are] a lot of considerations. I think no 1. was definitely like the space to grow, I think that was the biggest thing that came up I guess. I felt like I think over the past few months, I kind of realized, like how restricting the role can be and I feel like I want to do something more.”—50005_3

What they wanted to move on to remained within the domain of healthcare, and linked to harnessing lived experience, but was also, from their perspective, more impactful:

“Largely it is still PSS work, but I feel like I have reached a saturation point, and I think I shared this before, and for me it is more personal, than … yeah I think I have been thinking about it how I want to continue, and I mean I always want to contribute to peer support, but yeah I feel I have reached a point where, I mean I can still go on doing it and having sessions and working with the peers, but because where I am in my journey , I also feel like I want to move on to something else.”—50009_3

The tenth PSS who left the role over the course of the study did not have plans to continue his PSS career, but returned to his higher education.

## Discussion

We explored how the PSS role shifted over time and identified four distinct phases that characterized these shifts. Upon first entering the organization, the PSS often had never worked in multi-disciplinary teams and did not know what lay ahead. Despite receiving training designed to help them assume the role of PSS, perhaps insufficient efforts were made to prepare teams and new PSS for the introduction. Even when their role was established, their role remained fluid. Such a lack of clarity has been demonstrated in past studies to diminish happiness and increase animosity amongst the various team members (Otte et al., 2020). As a result, the lack of clarity needs to be addressed adequately to mitigate the development of adverse outcomes (Otte et al., 2020). The source of this role confusion might lay in the organization’s general definition of PSS employees, which was kept relatively non-specific to allow departments to integrate peers as they saw fit. Similarly, the certification and training may also lack specificity as it is designed to accommodate several forms of PSS work, including hospital- and community-based practice. This may echo results of previous assessments of the challenges of expanding peer services, which found training to be inadequate (Faulkner & Kalathil, 2012). One possible solution is the creation of an effective and impactful onboarding program. Newer PSS could be allowed to shadow senior PSS to improve the integration process. Working in pairs appears similarly beneficial to facilitate integration. It is also essential that existing staff be sufficiently made aware of the expected role of the PSS prior to their addition. Simple presentations and conversations packed into busy schedules may be insufficient, especially considering that some staff may see new PSS as service users first, and professionals second. Ensuring that existing staff are adequately prepared and receptive to PSS would ensure that provisions are made ahead of time to integrate them more effectively into the clinical teams.

Eventually, PSS were able to find a little niche within their respective care teams and discovered meaningful opportunities to support their clients. However, there still exists some ambiguity about the boundaries of their roles. A similar finding was also observed in another longitudinal study on youth mental health peer workers (Simmons et al., 2020). They highlighted the need for an ongoing review of the role the PSS plays within the broader care team. Such a requirement is essential when the organization into which the PSS is placed has a wide variety of sub-working-cultures. Such a review process could ensure that the role of the PSS is dynamic, and adjusted to meet the changing needs of the organization, yet still within the scope of the PSS capabilities. This could reduce the risks of job dissatisfaction observed in our study. Some peers mentioned that their role became too restrictive, consequently reducing their interest in the job. Another possible solution to this role dissatisfaction could be, as others have noted, multi-disciplinary teamwork. This teamwork does not entail generalizing the work of the PSS, but rather ensuring that the influence of the PSS can be shared equally with the team members and various professional groups. Staff retention may be increased by varying their task exposure to renew interest and ameliorate attention (White & Robinson, 2019). This should be done cautiously to prevent overstepping any existing professional boundaries, however.

Unfortunately, some PSS indicated their frustration and disappointment with the way they were being treated within the organization. They were not feeling particularly valued and often compared how they were being treated and respected to other members of the care team who seemed to hold higher positions within the unspoken hierarchy. Such a picture is similar to the situation in the US where a large number of PSS have given feedback on what they hoped for their future, which usually included additional education to advance their careers. These hopes were stifled by multiple barriers, most notably related to financial limitations (Jones et al., 2020). Unique issues related to leadership development concerning PSS progression have also been recently identified, and could pose a threat to the advancement of such potential career pathways (Jenkins et al., 2020). For example, the delicate balances that need to be achieved between multiple influences, such as stigma from co-workers, being a fellow peer, taking extra care of their mental health, and effective management of dual relationships are among some of the crucial factors to note.

### Implications

Our study is the first in Singapore and Asia to explore the way that PSS programs are implemented into mental healthcare institutions. From it we may derive specific PSS implications as well as wider human resource implications. Understanding the importance of clearly defining the PSS role will allow organizations to accurately market the job and provide prospective candidates with a clearer picture of the scope of their work. A clear understanding of the role will also help prepare new and existing staff to integrate. While it is normal that new employees experience a period of acclimatization, the flexibility required for such an acclimatization must not come at the expense of professional identity. Existing employees must be prepared to change to ensure the professional identities of incoming staff are respected, especially in the case of PSS where it may not be immediately apparent how best to integrate such a skillset into the clinical team. If the value of peer support work is not recognized, it may be too easy for existing staff to delegate their roles on a new employee.

Previous experiences staff may have had with PSS who left the role because of a deterioration of mental health might influence their willingness to work with new PSS. These experiences might represent a greater barrier to integration than the prejudices that might arise from total inexperience with PSS as colleagues. Therefore, managers may need to devote additional resources to assuaging worries, and may need to select a PSS willing to attempt to correct beliefs that arose from negative first-hand experiences with PSS.

Additionally, it is essential the PSS role is not overly restrictive and allows for the PSS to experience professional growth. Our participants mentioned something similar to recommendations made in parallel human resources research on the retention of staff in jobs with high turnover. Being exposed to multi-disciplinary team tasks might help staff retention because it allows the staff to experience a variety of duties, to a degree that respects professional boundaries while providing meaningful variety to the individual (White & Robinson, 2019). In this respect, setting hard boundaries around the PSS may hinder their long-term potential (Jones et al., 2020).

Perhaps most importantly, it is crucial to the success of any PSS program that organizations commit to making them feel as valued as any other mental health care professional. While this may not be a purely PSS-oriented suggestion, this concept of respect and being treasured by the organization was salient enough a point for us to highlight it as a driving factor that could elevate or hinder the growth of PSS programs.

Finally, understanding the trajectory of PSS may help organizations accommodate the various goals of the PSS as they emerge. The negative emotions that surfaced when PSS understood the limitations of their role represent an opportunity for support and career development. Organizations must decide if they choose to make provisions for the transition of PSS staff into other professional roles or if they prefer that the responsibility for career development remains in the individual. In some cases, peer support work may be a career, but in others it may be a transition. Exploring how the individual’s decision vis-à-vis this bifurcation emerges over their PSS tenure can help organizations build a valuable resource, especially if PSS retain the value of their lived experience and bring it into their new roles.

## Limitations

The pool from which we could select peer support specialist was limited, and therefore every effort was made to recruit all peers without appearing coercive. However, we have reason to believe those most likely to be dissatisfied with the role also were least likely to accept our offer of participation. We are therefore aware that a segment of the key informant pool could not be sampled. Furthermore, our sample of peers was obtained within a tertiary psychiatric hospital setting, and it is likely that the way their roles evolved would be different from peers who may be operating in community settings where the challenges and requirements of the role may differ.

While the number of potential participants was small, the variety of departments in which PSS were integrated was large. This introduces significant heterogeneity to the duties completed by PSS at the hospital. Ideally, we would have preferred to sample people performing similar duties in similar departments, but by virtue of the size of the population, we had to accept such heterogeneity and be satisfied with generating trajectories that could characterize the journeys of all our participants.

Concerning our chosen theoretical framework, implementation research is a rich source of theory that could have provided a different structure to the project. However, we chose to build the project on theory derived from fidelity-related work. This provided a subject-specific framework for looking at the various elements of the PSS role. The fidelity-related framework did provide a language which was familiar to the PSS, and allowed sufficient freedom to determine when and how the PSS began conducting PSS work that aligned with what has been identified in the literature as essential (Chinman et al., 2016). Choosing a more conventional framework from the implementation research might have produces results which fit more comfortably within that branch of research, but might not have led to the discovery of the negative impact of role narrowing.

## Conclusions

Peer support work is of great value, and greater attention needs to be placed on how PSS are integrated within organizations. As the cornerstone of a recovery-oriented approach, these individuals use their lived experiences to support and lift others who are similarly experiencing mental health conditions. Good-intentioned allowances for diversity of roles may be deleterious if insufficient guidance on task setting is given to those directly managing PSS. If initial duties are unclear, PSS experience role confusion. With time and experience, role clarity emerges. However, role narrowing may follow, which may eventually lead to PSS considering other professional roles. This transition may represent a path of career development and wider opportunity for people with lived experience to entre healthcare roles, taking with them the power of their lived experience. As the peer movement continues to grow in Singapore and globally, organizations must continue to strive for better integration of these essential members of a mental healthcare team.

## Appendix

### Qualitative Interview Guide

Can you tell me a bit about yourself? (First interview).

Tell me a bit about how you became a peer support specialist? (First interview).

Tell me a bit about why you became a peer support specialist. Was there a turning point after which you wanted to be a peer? (First interview).

Can you tell me a bit about what has changed since you last saw us? (Second, thrid interview).

Can you take me through a regular day?

- What are you regular activities?
- What are the challenges of your daily work?
- What are the rewards?

Can you tell me about the process you engage with as a peer to help service users?

Do you teach any skills to your service users?

Can you tell me about what it is like to be part of your team?

- What were the challenges to integrating peers into the teams?
- What made your integration into existing services easier?

Can you tell me about the way in which you have to document your activities?

Can you tell me a bit about your co-workers and the way you collaborate?

What has the management been like? Have they been supportive?

Can you think of anything that improved your skills?
